# NMR-Based Metabolomics in Investigation of the Radiation Induced Changes in Blood Serum of Head and Neck Cancer Patients and Its Correlation with the Tissue Volumes Exposed to the Particulate Doses

**DOI:** 10.3390/ijms22126310

**Published:** 2021-06-11

**Authors:** Łukasz Boguszewicz, Agata Bieleń, Mateusz Ciszek, Jacek Wendykier, Krzysztof Szczepanik, Agnieszka Skorupa, Jolanta Mrochem-Kwarciak, Krzysztof Składowski, Maria Sokół

**Affiliations:** 1 Department of Medical Physics, Maria Sklodowska-Curie National Research Institute of Oncology, Gliwice Branch, 44-102 Gliwice, Poland; mateusz.ciszek@io.gliwice.pl (M.C.); agnieszka.skorupa@io.gliwice.pl (A.S.); maria.sokol@io.gliwice.pl (M.S.); 2 1st Radiation and Clinical Oncology Department, Maria Sklodowska-Curie National Research Institute of Oncology, Gliwice Branch, 44-102 Gliwice, Poland; agata.bielen@io.gliwice.pl (A.B.); krzysztof.skladowski@io.gliwice.pl (K.S.); 3 Radiotherapy Planning Department, Maria Sklodowska-Curie National Research Institute of Oncology, Gliwice Branch, 44-102 Gliwice, Poland; jacek.wendykier@io.gliwice.pl; 4 Radiotherapy Department, Maria Sklodowska-Curie National Research Institute of Oncology, Gliwice Branch, 44-102 Gliwice, Poland; krzysztof.szczepanik@io.gliwice.pl; 5 Analytics and Clinical Biochemistry Department, Maria Sklodowska-Curie National Research Institute of Oncology, Gliwice Branch, 44-102 Gliwice, Poland; jolanta.mrochem-kwarciak@io.gliwice.pl

**Keywords:** metabolomics, cancer, radiotherapy, toxicity, NMR

## Abstract

In the present study, we analyze the nuclear magnetic resonance (NMR) blood serum metabolic profiles of 106 head and neck squamous cell carcinoma (HNSCC) patients during radio (RT) and concurrent radio-chemotherapy (CHRT). Four different fractionation schemes were compared. The blood samples were collected weekly, from the day before the treatment until the last week of CHRT/RT. The NMR spectra were acquired on A Bruker 400 MHz spectrometer at 310 K and analyzed using multivariate methods. Seven metabolites were found significantly to be altered solely by radiotherapy: N-acetyl-glycoprotein (NAG), N-acetylcysteine, glycerol, glycolate and the lipids at 0.9, 1.3 and 3.2 ppm. The NMR results were correlated with the tissue volumes receiving a particular dose of radiation. The influence of the irradiated volume on the metabolic profile is weak and mainly limited to sparse correlations with the inflammatory markers, creatinine and the lymphocyte count in RT and the branched-chain amino-acids in CHRT. This is probably due to the optimal planning and delivery of radiotherapy improving sparing of the surrounding normal tissues and minimizing the differences between the patients (caused by the tumor location and size).

## 1. Introduction

The anatomical sub-sites and the morphological sub-varieties of head and neck squamous cell carcinomas (HNSCC) have different biology, patterns of spread, and treatment requirements [1]. Their therapeutic irradiation remains a challenge for the radiation oncologists and the survival rates for locally advanced HNSCC are still poor [2]. 

Over the last few decades there has been a tremendous development in radiation therapy (RT) associated with the introduction of advanced dose-delivery techniques, including intensity-modulated radiation therapy (IMRT) [3]. In parallel, various altered fractionation strategies, e.g., hyper-fractionation, accelerated fractionation, and hypo-fractionation have been introduced and demonstrated improvements in overall and progression-free survival when compared with conventional fractionation radiotherapy [4]. 

Treatment intensification by applying RT alone or in combination with chemotherapy (CHRT), has improved the survival of the patients with HNSCC, but factors like large irradiated volumes, use of chemotherapy, bilateral radiation and dose intensification may increase the toxicity even with advanced techniques [5,6,7]. The significant problems associated with a high toxicity as well as the resistance to current treatments highlight an urgent need for more effective therapies, alongside clinically relevant biomarkers to stratify patients and improve the treatment outcomes [8].

The RT induced toxicity in HNSCC, also in a predictive perspective, is being extensively studied at clinical [9,10,11] as well as metabolic [12,13] and proteomic levels [14,15,16]. In our previous works we identified the preliminary candidates for the toxicity biomarkers in the sera of the HNSCC patients using NMR-based metabolomics. The metabolites involved in the inflammatory processes, energy metabolism and disturbed membrane metabolism were found to be significantly correlated with acute radiation-associated toxicity [12], while a marked increase in the concentration of the ketone bodies was found to be a strong predictor of cachexia [13].

Very recent studies on radiation biodosimetry report that it is possible to distinguish the metabolic changes in blood and urine due to a partial- and total-body irradiation of animals and humans [17,18]. However, in case of tumors of a similar stage and location, e.g., in the locally advanced head and neck cancers, the irradiated volumes differ only slightly. Therefore, it may be much more difficult to observe the metabolic changes correlated with the size of the irradiated area.

The study is based on the hypotheses that the alterations in the blood serum metabolic profiles reflect the injury to the tumor and normal tissue during the radio- or chemoradiotherapy of the HNSCC patients and that these effects can be distinguished via an NMR-based metabolomics as well as that the metabolic response is proportional to the irradiated volume, even within a particular treatment scheme. The metabolic response to the therapy is analyzed and correlated in a week to week manner with the tissue volumes receiving a particular dose of radiation. The aim of the study is to descriptively characterize the metabolic response to anti-cancer treatment which may aid in understanding of the background of the cancer molecular mechanism, acute radiation-associated toxicity intensification in some patients as well as being able to contribute to a more personalized approach to the treatment.

## 2. Materials and Methods

### 2.1. Characteristics of the Patients Groups

The retrospective study was approved by the Ethics Committee and the informed written consent of the participants was obtained. The studied group consisted of 106 HNSCC patients, 79 men and 27 women, all Caucasians, aged between 41 and 79 years and treated in the 1st Radiation and Clinical Oncology Department of Maria Sklodowska-Curie National Research Institute of Oncology, Gliwice Branch, Poland. All patients were treated with radical intent with radiotherapy (RT) or concurrent radio-chemotherapy (CHRT) using four fractionation techniques [12]:

- CONV (conventional fractionation): 2 Gy per fraction, 35 fractions, the total dose 70 Gy, delivered once-a-day and 5-days-a-week with a weekend break, for 7 weeks; 55 patients treated with a concurrent CHRT, 7 patients treated with RT only;
- CAIR (continuous accelerated irradiation): 1.8 Gy per fraction, 40 fractions, the total dose 72 Gy, delivered once-a-day and 7-days-a-week, for 6 weeks; 22 patients.
- Manchester scheme (accelerated hypofractionated irradiation): 3 Gy per fraction, 17 fractions, the total dose 51 Gy, delivered once-a-day and 5-days-a-week with a weekend break, for 3.5 weeks; 19 patients.
- SIB (accelerated irradiation with simultaneous integrated boost): 2.2 Gy per fraction to the total dose 66 Gy for gross tumor volume (PTV1), 2.0 Gy per fraction to the total dose 60 Gy for gross tumor volume plus anatomical margins (PTV2), 1.8 Gy per fraction to the total dose 54 Gy for elective fields (PTV3), all in 30 fractions, delivered once-a-day and 5-days-a-week with a weekend break, for 6 weeks; 3 patients.

The detailed patients’ characteristics are presented in Table S1 (Supplementary Materials).

### 2.2. Volumes Receiving a Particular Dose of Irradiation

The volumes receiving the following doses: 5, 10, 15, 20, 25, 30, 40, 50, 60 and 70 Gy, were calculated with the Varian treatment planning system Eclipse v.16 using the “Convert Isodose Level to Structure” functionality. The volumes of the obtained structures were calculated using “Measure Volume” option. The distribution of the isodoses in the irradiated target depended on the tumor size and location, a method of normalization of the distribution, protection of critical organs as well as preferences of the planning medical physicist.

### 2.3. Serum Samples Collection

The overnight fasting blood samples from the peripheral vein were collected weekly, starting from the day before the treatment and stopping within the last week of the RT/CHRT completion, resulting in a total number of 738 blood samples. The samples were incubated for 30 min at room temperature and then centrifuged (1000× *g*, 10 min) to remove the clot, and stored frozen at −80 °C until the NMR measurements were performed.

### 2.4. Sample Preparation for NMR Spectroscopy

The serum samples were thawed in two steps (at 4 °C and at room temperature) and mixed with a phosphate buffer (pH 7.4) containing D_2_O and TSP. The aliquots of 600 µL of the solution were transferred into 5 mm Wilmad WG-1235-7 NMR tubes (Wilmad Labglass, Vineland, NJ, USA) and kept at 4 °C until the NMR analysis. 

### 2.5. Measurement Protocol

The same measurement protocol as in our previous metabolomic studies [12,13] was applied. The ^1^H NMR spectra were acquired on a Bruker 400 MHz Avance III spectrometer (Bruker Biospin, Rheinstetten, Germany) equipped with a 5 mm PABBI probe. The quality control tests were performed at every measurement day. The NMR probe tuning and matching, shimming, determination of the transmitter offset value for the water pulse presaturation and 90° pulse adjustments were always made for each sample. The receiver gain was set to 90.5 and the temperature to 310 K for all the experiments. For each serum sample, four ^1^H NMR spectra were acquired with different pulse sequences: Nuclear Overhauser Effect SpectroscopY (NOESY), Carr–Purcell–Meiboom–Gill (CPMG), diffusion edited (DIFF) and J-resolved (JRES). The characteristics of the spectra as well as the pulse sequence parameters are given in the Supplementary Materials (Table S2) .

### 2.6. Spectra Post-Processing

All 1D spectra were processed with a line broadening of 0.3 Hz and automatically phase corrected (in Topspin software from Bruker Biospin), referenced to the methyl doublet of alanine at 1.5 ppm and bucketed over the region 9.0–0.5 ppm with the bucket width set to 0.002 ppm using AMIX software (Bruker Biospin). The water signal region (5.15–4.38 ppm, d = 0.77 ppm) was excluded, as the water residual signal after suppression is not of interest and often interferes with the signals from other metabolites. No normalization was applied. This is the standard processing protocol used in our metabolomic lab [12,13].

### 2.7. Metabolite Identification

The metabolite identification was performed based on the comparisons with the reference compounds library (in Chenomx NMR Suite Professional (Chenomx Inc., Edmonton, AB, Canada)) using the CPMG spectra, as well as on the basis of the multiplicity and scalar couplings information extracted from the 2D JRES spectra, and using the information from the Human Metabolome Database (http://www.hmdb.ca/ accessed on 6 June 2021) and available literature.

### 2.8. Metabolite Quantification

The low molecular weight metabolites were quantified based on the 1D positive projections of the JRES spectra. The diffusion edited spectra were used for quantification of the lipid signals. The integrals were measured in the spectral regions defined individually for each metabolite using “the sum all points in region” method in AMIX (Bruker Biospin) software.

### 2.9. Data Analysis and the Validation of the Multivariate Model

The multivariate analyses were performed on the 1D positive projections of the JRES spectra using SIMCA-P+ (Umetrics, v. 15) software. The NMR variables were Pareto scaled. Orthogonal partial least squares discriminant analysis (OPLS-DA) was used for class discrimination. The OPLS-DA models were assessed using permutation testing and ANOVA of the cross-validated residuals (cv-ANOVA). The detailed description and interpretation of the OPLS-DA plots is available in [12]. The univariate statistical analyses, e.g., the Spearman’s correlation, were carried out using Statistica software (Statsoft, v. 12).

## 3. Results

### 3.1. Differences in Volumes Receiving Particular Dose of Irradiation

**Figure 1:**
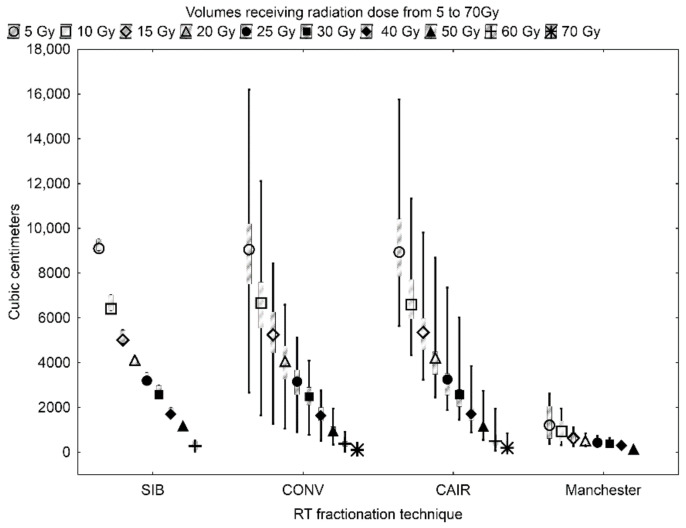
The box and whisker plot comparison of the volumes (in cm^3^) receiving a particular radiation dose: from 5 to 70 Gy in SIB, CONV and CAIR fractionated radiotherapy (RT); and from 5 to 50 Gy in Manchester fractionated RT.

The RT fractionation strategies were compared taking into account the volumes receiving the same dose. Figure 1 shows the box-plot of the volumes receiving from 5 to 70 Gy for the applied RT techniques. It is clearly seen that the volumes receiving a particular dose are distinctly smaller for the patients treated with the Manchester technique. The statistical significance of the observed difference was confirmed using the Kruskal–Wallis ANOVA tests with *p*-value < 0.05. There were no significant differences between the remaining RT techniques, except the volumes receiving 50 and 70 Gy, which were significantly larger in the CAIR technique when compared to CONV.

### 3.2. Blood Serum Metabolic Profile vs. Radiation Therapy

In order to eliminate a possible influence of the differences in the volumes receiving a particular RT dose as well as of those in the fraction dose, the following analyses were performed, separately for the patients treated with:

- Concurrent CHRT (CONV RT fractionation with one to three cycles of CHT administered at weeks 0, 3 and 6 during RT),
- RT with CAIR/CONV/SIB fractionation,
- RT with Manchester fractionation.

Two types of OLS-DA analyses were performed for each of the three studied groups:

- Type I—the metabolic profiles were compared in a weekly increment in RT (e.g., week-0 vs. week-1, week-1 vs. week-2, etc.).
- Type II—the changes in the blood serum during RT were compared to week-0 (e.g., week-0 vs. week-1, week-0 vs. week-2, etc.).

The OPLS-DA method was not able to deliver significant models differentiating the metabolic changes between the adjacent weeks (Type I analyses) as well as significant models showing early response to RT (up to the second week) except CHRT where all the Type II OPLS-DA models were significant. The results below show only the statistically important models.

#### 3.2.1. Concurrent CHRT

**Figure 2:**
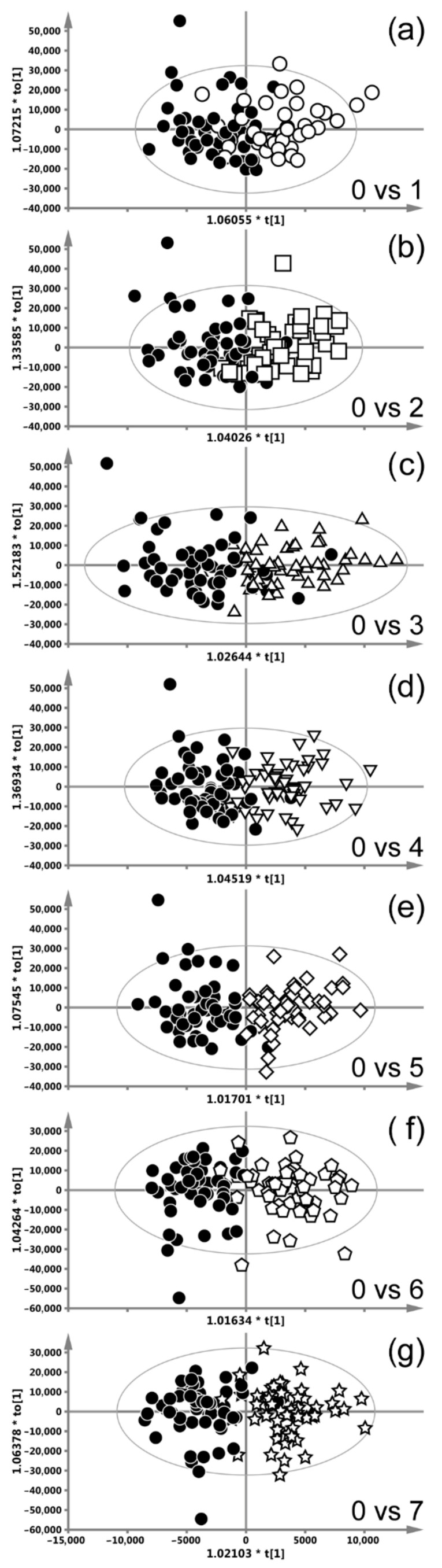
OPLS-DA scores plots differentiating week-0 from the consecutive weeks of radio-chemotherapy (CHRT), the x-scale (predictive direction t [1]) is the same for all plots. Week-0 vs. week-1 (**a**), week-2 (**b**), week-3 (**c**), week-4 (**d**), week-5 (**e**), week-6 (**f**) and week-7 (**g**).

Figure 2 shows the OPLS-DA score plots differentiating week-0 from the consecutive weeks of the CHRT treatment, the x-scale (predictive direction t [1]) is the same for all the plots. As the treatment progresses, there is a visible improvement in the separation of the particular groups. The corresponding s-line plots, based on which the important metabolites have been identified, are available in the Supplementary Materials (Figure S1). The highest number of the significantly treatment-altered metabolites was identified by the OPLS-DA model separating the weeks 0 and 4. The summary of the OPLS-DA analyses with the models’ diagnostic parameters as well as a complete list of the metabolites that are significantly disturbed throughout the treatment (week-1 to week-7) compared to the baseline (week-0) levels are provided in Table 1. The initial (week-1) response to the treatment is manifested by the simultaneously increased levels of the branched-chain amino acids (BCAAs: leucine, valine and isoleucine), N-acetyl-glycoprotein (NAG), N-acetylcysteine, glutamine, creatinine and tyrosine as well as the decreased lipid signals at 3.2 ppm and the deformations (decrease) of the downfield part of the resonance at 0.9 ppm. The lipid band at 0.9 ppm derives from the terminal CH_3_ groups of fatty acids, triglycerides and phospholipids as well as from the cholesterol methyls [19,20]. Thus, the changes in its shape and intensity indicate the variation in the mutual contributions of the individual components. Week-2 brings about a normalization of the observed changes, except the decrease in the lipids at 3.2 and 0.9 ppm. However, by week 3, a part of the initial treatment-induced alterations recurs and persists throughout the further treatment, i.e., the increases in NAG and N-acetylcysteine. Furthermore, the levels of glycerol and glycolate are observed to increase. The temporarily elevated amino acids are BCAAs and glutamine (increase at week-4), the lipid resonance at 1.3 ppm (increase in the upfield part of the band at weeks 6 and 7), whereas betaine (week 7), glucose (week 6) and methanol (weeks 3, 5 and 7) become temporarily reduced at the indicated time points.

#### 3.2.2. Radiotherapy with CAIR/CONV/SIB Fractionation

**Figure 3:**
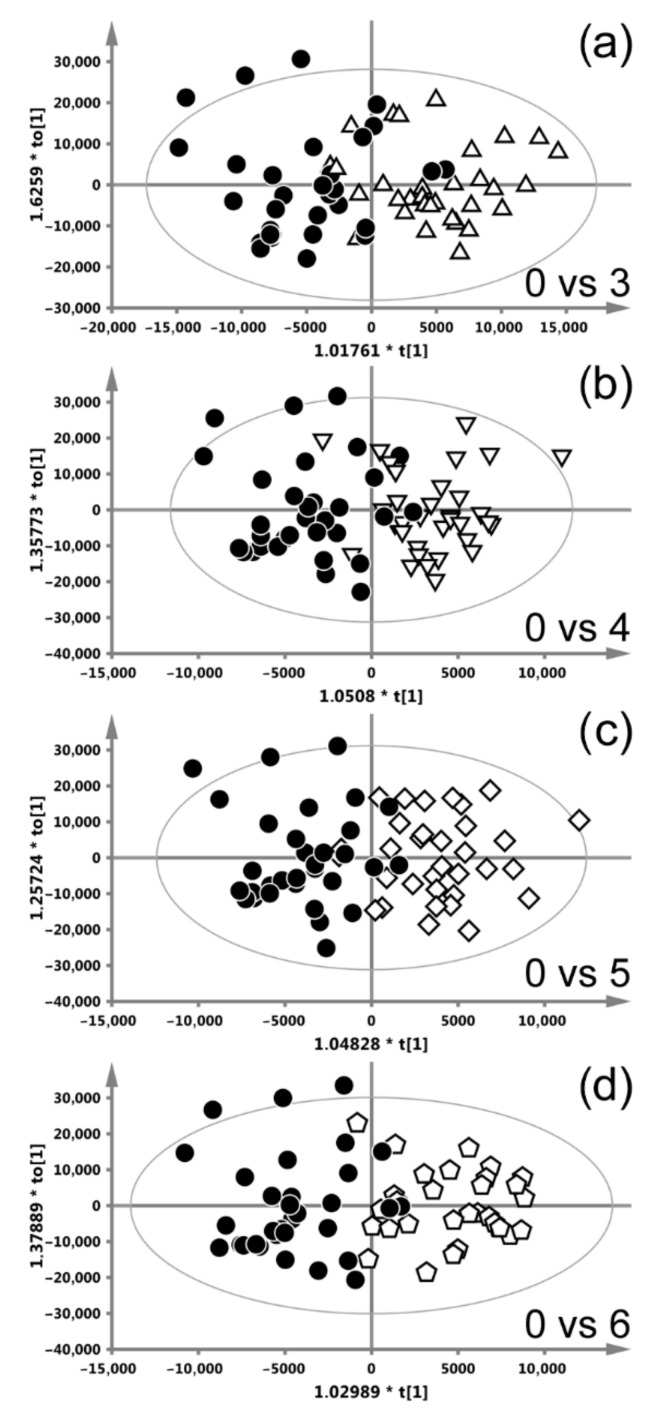
OPLS-DA score plots differentiating week-0 from the consecutive weeks of radiotherapy (RT) with CAIR/CONV/SIB fractionation. Week-0 vs. week-3 (**a**), week-4 (**b**), week-5 (**c**) and week-6 (**d**). The models differentiating the remaining weeks from week 0 were not significant.

Figure 3 shows the OPLS-DA score plots differentiating week-0 from weeks 3–6 (the models differentiating the remaining weeks were insignificant) of RT with CAIR/CONV/SIB fractionation. The presented plots show a decent class separation; the corresponding s-line plots, based on which the important metabolites have been identified, are available in the Supplementary Materials (Figure S2). Table 2 summarizes the OPLS-DA results. The metabolic response to RT is similar throughout the treatment, with the highest number of altered metabolites at week 6, exhibiting an increase in NAG, N-acetylcysteine, glycerol, glycolate and a decrease in alanine, creatinine, betaine, the lipids at 1.3, 3.2 and 5.3 ppm as well as a decrease in the upfield part of the lipid band at 0.9 ppm.

#### 3.2.3. Radiotherapy with Manchester Fractionation

**Figure 4:**
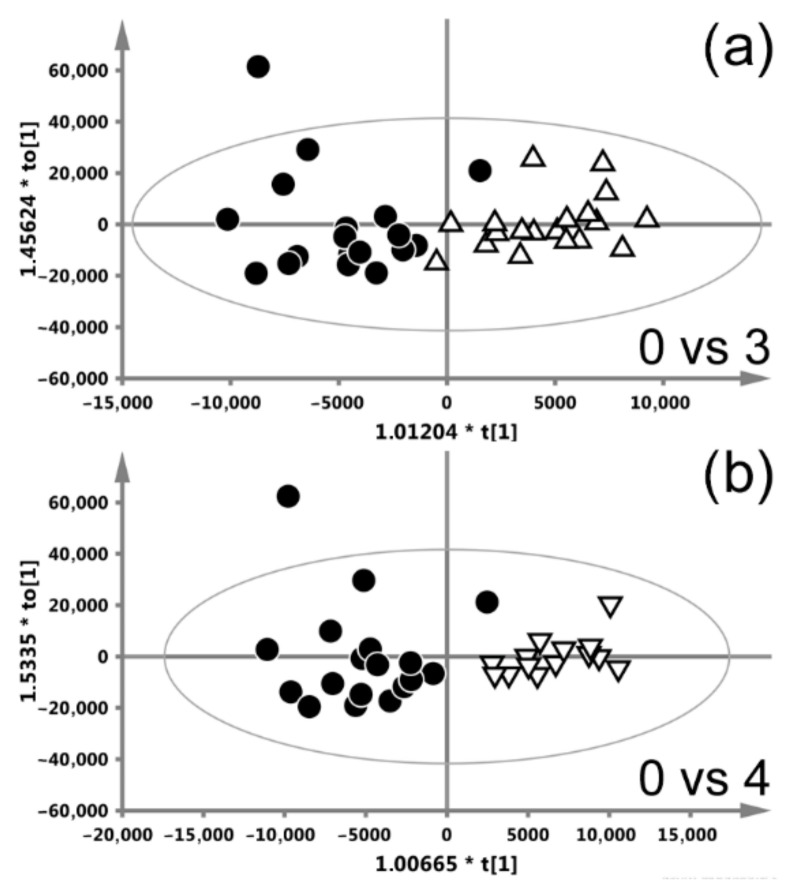
OPLS-DA scores plots differentiating week-0 from the week-3 (**a**) and week-4 (**b**) of radiotherapy (RT) with Manchester fractionation. The models differentiating week-0 from week-1 and week-2 were not significant.

Figure 4 shows the OPLS-DA scores plots differentiating week-0 from week-3 and week-4 (the models differentiating week-1 and week-2 from week-0 were not significant) of RT with Manchester fractionation. The classes are separated clearly and the corresponding s-line plots (based on which the important metabolites have been identified) are available in the Supplementary Materials (Figure S3). The summary of the OPLS-DA analyses is presented in Table 3. The metabolic response to RT in week-3 and week-4 comprises the increases in NAG, N-acetylcysteine, glycerol and glycolate as well as a reduction in the lipids at 1.3 ppm and the lipids contributing to the upfield part of the bands at 0.9 and 3.2 ppm. Furthermore, at week-4 the levels of glutamine and lysine are higher compared to week-0.

#### 3.2.4. Irradiated Volume Impact on the Metabolic Profile

**Figure 5:**
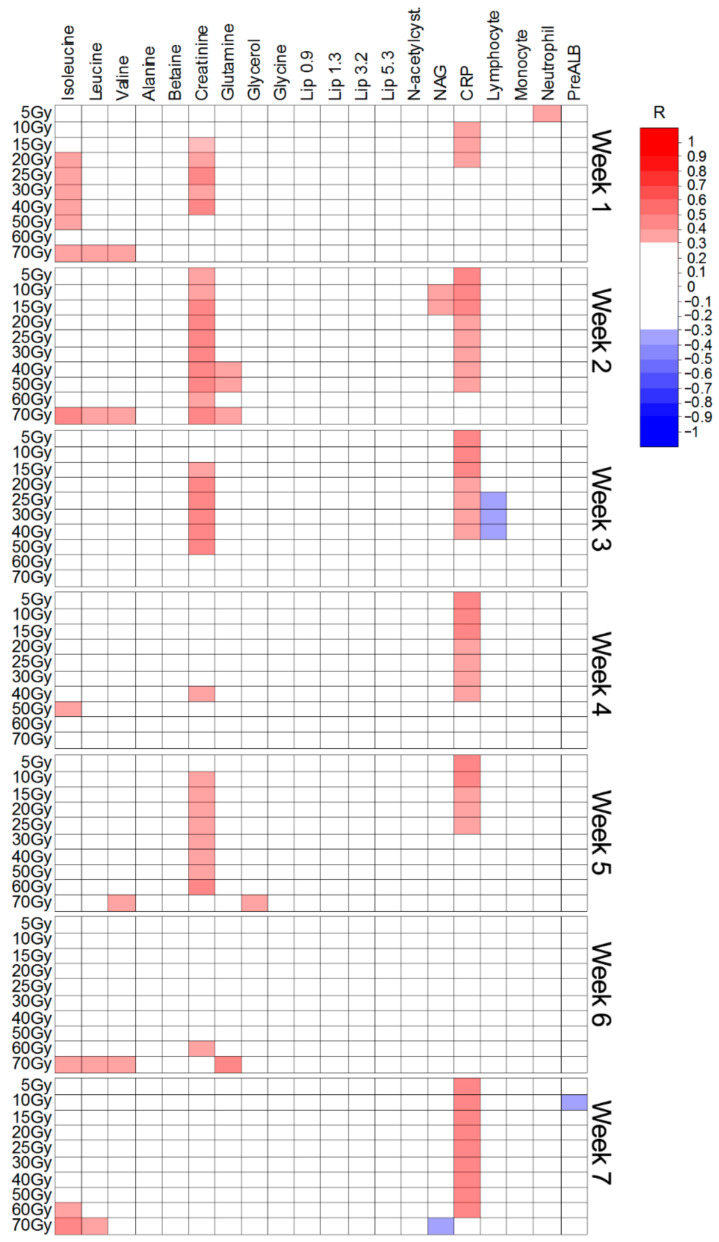
Heat map of the Spearman’s correlations between the blood serum metabolites and the irradiated volumes receiving a particular dose during concurrent chemoradiotherapy.

**Figure 6:**
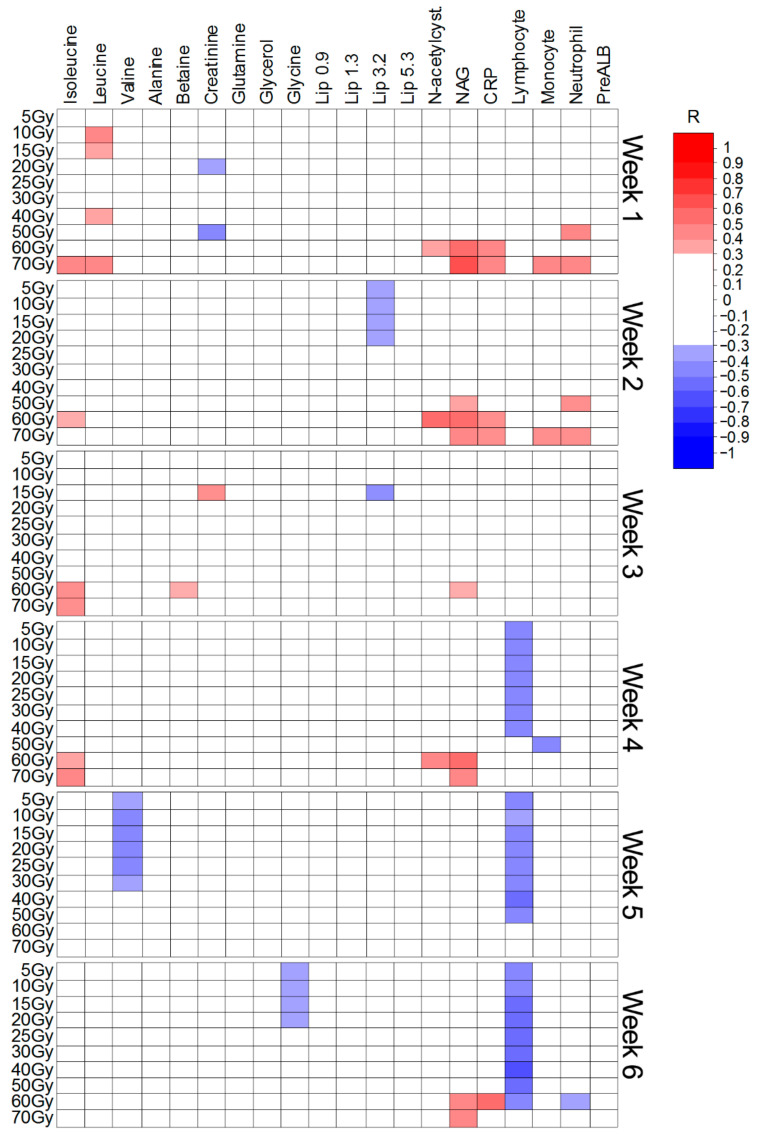
Heat map of the Spearman’s correlations between the blood serum metabolites and the irradiated volumes receiving a particular dose during CAIR/CONV/SIB fractionated radiotherapy.

**Figure 7:**
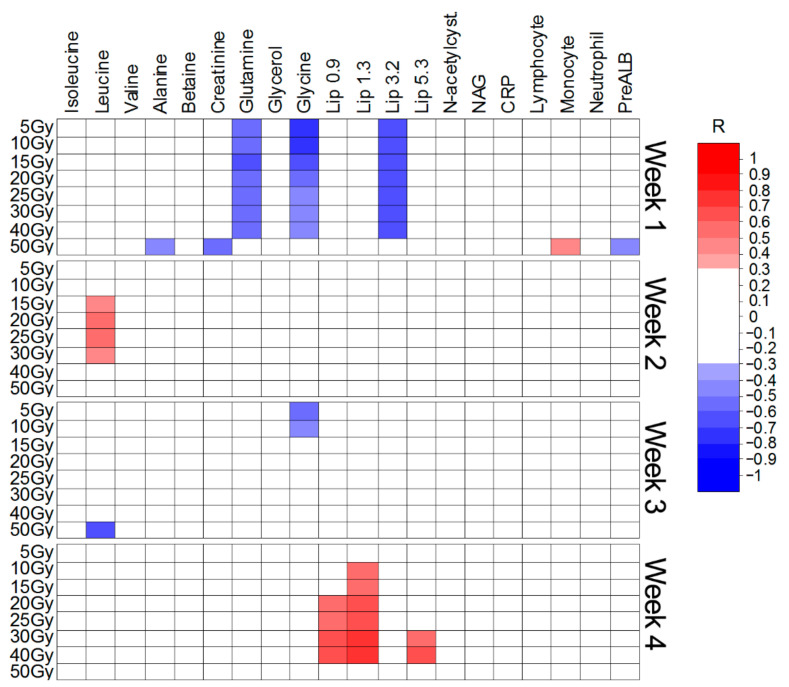
Heat map of the Spearman’s correlations between the blood serum metabolites and the irradiated volumes receiving a particular dose during Manchester fractionated radiotherapy.

The Spearman’s rank correlation coefficients (R) were calculated to examine the relationship between the intensities of the twenty important metabolites (Table 1, Table 2 and Table 3) and the irradiated volumes. The analyses were performed separately for each week of the treatment. In addition, the correlations were also assessed for five laboratory parameters, often analyzed during RT, i.e., c-reactive protein (CRP), lymphocyte count, monocyte count, absolute neutrophil count and the prealbumin levels (PreALB). Only the significant (with the threshold set at *p* < 0.05 and R ≥ 0.30) results are presented. Figure 5, Figure 6 and Figure 7 show the Spearman’s correlation heat maps obtained for CHRT (Figure 5), CAIR/CONV/SIB radiotherapy (Figure 6) and radiotherapy with Manchester fractionation (Figure 7). The patterns of the significant correlations obtained for the studied treatment modalities differ and, although the significant correlations are observed for 16 (out of 20 identified in the OPLS-DA analyses) metabolites, the overall number of significantly correlated changes in the treatment course is relatively small and show high variability, even within a particular treatment modality. The most frequent correlations between the blood serum metabolites and the irradiated volumes involve the branched chain amino-acids (BCAA: isoleucine, leucine, valine), creatinine, NAG and lipids (in Manchester fractionation). Moreover, for the analyzed clinical parameters, the correlations with the irradiated volumes are mostly observed for CRP and the lymphocyte count.

In concurrent chemoradiotherapy (Table 1 and Figure 5), from the metabolites elevated during the treatment, only the increase in BCAAs is positively correlated with the irradiated volume. Such a correlation is also observed for creatinine; however, this metabolite was significantly increased only when comparing week-0 and week-1 of the treatment. Surprisingly, NAG (NMR marker of inflammation) shows almost no correlation with the irradiated volume, contrary to CRP. None of the metabolites decreasing during the treatment were found to correlate with the irradiated volume.

The increase in BCAAs is also positively correlated with the irradiated volume in CAIR/CONV/SIB radiotherapy (Figure 6); however, this elevation is not significant (Table 2). In turn, the increase in the NAG signal during the RT course (and in CRP during the first two weeks of RT) shows a significant positive correlation with the volumes receiving 60 and 70 Gy (Figure 6). Among the metabolites decreasing during the treatment, creatinine and lipids show a sparse negative correlation with the irradiated volume. A marked negative correlation is observed between the lymphocyte count and the volumes receiving from 5 to 60 Gy (weeks 4, 5 and 6) (Figure 6).

The correlations heat map for Manchester fractionated RT (Figure 7) shows a pattern, which does not correspond well with the OPLS-DA analyses (Table 3). The distinct negative correlations between glutamine, glycine, the lipid signals at 3.2 ppm and the volumes receiving from 5 to 40 Gy are observed after week-1 of RT. However, none of these metabolites are significantly changed at the early stage of the treatment (Table 3). At week-4 the lipid signals positively correlate with the volumes receiving from 10 to 40 Gy (the lipids at 1.3 ppm), from 20 to 40 Gy (the lipids at 0.9 ppm) and from 30 to 40 Gy (the lipids at 5.3 ppm). This is in contrast to the results from OPLS-DA, where the lipid signals (except the lipids at 5.3 ppm) are decreased due to RT.

## 4. Discussion

Radiation-induced tissue injury is a major dose-limiting toxicity in head and neck cancer patients. The incidence of such events increases with the dose and the irradiated volume. In the present study, we focused on the HNSCC patients treated with RT or CHRT and analyzed the changes in their serum metabolic profiles in relation to the irradiated volume in the head and neck area. For the purpose of the present study, 10 volumes receiving the doses of 5, 10, 15, 20, 25, 30, 40, 50, 60 and 70 Gy, were calculated for each patient using the treatment planning system (for the Manchester fractionation only eight volumes were obtained as the maximum dose was 51 Gy). As seen in Figure 1 the volumes receiving a particular dose are similar for CONV, CAIR and SIB RT, but for Manchester fractionation they are significantly smaller. In the group treated according to the Manchester schedule there were only the patients with low stage (T1N0M0 and T2N0M0) larynx cancers, and thus with very small malignant lesions. In order to exclude the potential biasing factors, the analyses for the patients treated with CHRT, Manchester fractionation and with CAIR/CONV/SIB fractionation were performed separately.

In our previous study [13] we observed the intense signals of the ketone bodies (3-hydroxybutyrate, acetoacetate and acetone) in the NMR spectra of the HNSCC patients developing cachexia during RT/CHRT. These signals (except acetone) are not normally detected in the blood serum, while in case of malnutrition and cachexia they may dominate the NMR spectra [13]. Thus, the spectral regions corresponding to these signals were excluded from the analyses in order to detect mainly the changes due to radiotherapy.

Regardless of the applied RT fractionation, the OPLS-DA method was not able to find the metabolic features distinctive for the early therapy stages as well as the features separating the consecutive treatment weeks (the statistically significant differences were observed only for the OPLS-DA model differentiating week-0 and week-1 of CHRT). There are patient- and blood collection procedure-related groups of the reasons responsible for these difficulties. In the patient-related group, the inter-individual differences in radio-sensitivity, being due to various endogenous and exogenous factors, are of importance. Thus, the response to ionizing radiation is individual and variable, being influenced by age, smoking, diabetes, collagen vascular disease, genotype [21] and the drugs taken [22], which in consequence weakens the power of the statistical tests [13]. In turn, the other group includes the causes related mainly to the inaccuracies in the blood sampling procedure. The weekly schedule of the blood collecting was not always followed precisely (due to the difficulties resulting from a daily hospital work); when the intervals between the blood sampling time-points were too long (2–4 days), such samples were excluded from the analysis, to minimize this variability, or assigned to the previous or following week. Moreover, it cannot be excluded that the weekly interval may be too short to observe a significant systemic metabolic response to irradiation. This supposition finds additional support in the subsequent analyses, where the consecutive weeks of the treatment were compared to week-0. In case of radiotherapy alone (Table 2 and Table 3 and Figure 3 and Figure 4) no significant differences were found until comparing week-0 with week-3. The OPLS-DA models differentiating week-0 from week-1 and week-2 in CHRT were significant, but the observed metabolic alterations may be influenced by or due to chemotherapy, which was administered at weeks 0, 3 and 6.

Of the 20 metabolites identified by the OPLS-DA models as important for the discrimination between the respective weeks of the treatment and week-0 (Table 1, Table 2 and Table 3), only seven are important in all treatment groups (CHRT, CAIR/SIB/CONV and Manchester): NAG, N-acetylcysteine, glycerol, glycolate and the lipids at 0.9, 1.3 and 3.2 ppm. Figure S4 (Supplementary Materials) shows a summary of the OPLS-DA analyses; the features common to the particular treatment methods are color-coded. The lipid signals are from the CH_2_ and CH_3_ groups of cholesterol and cholesteryl ester (0.9 ppm), the fatty acyl–(CH_2_)_n_ chains (the line centered at 1.3 ppm) and the (CH_3_)_3_ sphingomyelin head groups (a singlet at approximately 3.2 ppm).

The NAG signal (centered at approximately 2.06 ppm) is chemically nonspecific in origin, coming from the N-acetyl methyl group resonances from a subset of mobile N-acetylglucosamine (GlcNAc) residues on the glycan branches of abundant glycoproteins. It was called by Otvos et al. as GlycA (glycoprotein acetylation) [23]. Recently, the simultaneous measurement of up to three signals associated to glycoproteins, such as GlycB (concentration of acetyl groups of N-acetylneuraminic acid) and GlycF (concentration of acetyl groups of N-acetyl-sugars unbonded to proteins) has been proposed, beyond GlycA, to improve accuracy and potency to this biomarker [24]. Actually, this biomarker works even better than C-reactive protein, a widely used classic inflammatory biomarker. NAGs, mainly N-acetylglucosamine and N-acetylneuramic acid, are acute phase proteins with anti-inflammatory properties and are expressed more during inflammation and immune responses [25,26]. When studying the head and neck cancer treatment toxicity, NAG was observed to increase during RT/CHRT [13] and this increase was correlated with acute radiation-associated toxicity [12] and significantly positively correlated with CRP. However, both these markers are claimed to capture the different aspects of the inflammatory response [27], as their half-lives differ [28].

The effect of RT on inflammation and the immune response is dictated by the radiation type, dose, dose rate, intensity/fractionation, delivery method, field size, and the total cumulative dose. It initiates and influences the inflammatory/immune system in the tumor microenvironment and modulates immune cell populations [29]. N-acetylcysteine (N-acetyl derivative of the amino acid L-cysteine) is well known from being involved in antioxidant and anti-inflammatory (through inhibiting the proinflammatory cytokines) processes [30,31,32]. N-acetylcysteine is synthesized endogenously [33], as well as being widely used as a medication or a dietary supplement [30]. We may hypothesize that the observed increase in the blood serum levels of N-acetylcysteine during RT is, similarly to NAG, related to the inflammation processes. Glycerol is the important component of phospholipids and triglycerides and is released into the bloodstream (together with the ketone bodies) during fasting [34,35]. Anticancer treatment in the head and neck area often leads to temporal, yet significant, pain, xerostomia, dysphagia and dysgeusia. These side effects result in a prolonged fasting and a rapid weight loss in HNSCC patients during RT/CHRT [13]. Thus, the increase in the glycerol signal, though much less intense than of the signals of the ketone bodies (removed from the spectra for the sake of improving the visibility of the minor changes), presumably results from the fasting and ketosis. In fact, in the studied group glycerol shows a significant positive correlation with the ketone bodies signals with the R values of 0.5, 0.45 and 0.44 for 3-hydroxybutyrate, acetoacetate and acetone, respectively.

There is no well-established evidence in the literature on the direct connection between the glycolate blood levels and RT. The glycolate concentration in the cells is regulated, inter alia, by glyoxal metabolism via the glyoxalase system, frequently being overlooked in cancer research [36]. Its enzymes, the glyoxalase I (Glo1) and glyoxalase II (Glo2), are glutathione (GSH)-dependent. Glo1 catalyzes the GSH-dependent removal of the endogenous reactive dicarbonyl metabolite, methylglyoxal, formed mainly as a side product of anaerobic glycolysis [37]. Glo2 catalyzes the conversion of S-d-lactoylglutathione to d-lactate and reforms GSH consumed in the Glo1-catalysed reaction step [38]. Since both are involved in antioxidative defense, RT-induced influence on the functioning of this system may be of importance from the point of view of the therapy effectiveness. Glo1 was found to be increased in erythrocytes of the patients following radiotherapy for larynx cancers [39]. On the other hand, an inhibition of Glo1 was observed in the irradiated breast cancer cells [40]. Furthermore, an increase in glyoxylate (a metabolite produced through the oxidation of glycolate [38]) was observed in the urine of the irradiated rats with the chromatographic methods for metabolomics [41]. Thus, it may be speculated that the increased levels of glycolate observed by us from week-3 until the end of RT correspond to the mentioned processes. Obviously, such supposition requires further investigation and implementation of additional metabolomic tools.

In contrast to glycolate, the effects of ionization radiation on the serum lipids are well documented in various types of cancer, like prostate [42], breast [43] and head and neck [13,44] cancers, and are explained in terms of the tumor response to the treatment as well as choline-related signaling.

In CHRT as well as in CAIR/CONV/SIB radiotherapy at the final stage of the treatment (week-5 and week-6), we observed a significant reduction in alanine (one of the major substrates for gluconeogenesis) (Table 1 and Table 2). A decrease in the alanine signal was found to correlate with weight loss in the HNSCC patients undergoing RT/CHRT [13], and the lowered post-RT alanine signals were observed in the patients with high acute radiation-associated toxicity [12]. The homogeneous group of the patients with low stage larynx cancers and very small irradiated volumes (Figure 1) treated with Manchester RT (Table S1) presents a weaker acute radiation-associated toxicity compared to the CHRT and CAIR/CONV/SIB groups and a significant weight loss in this group is rarely observed [13]. A better status of the Manchester group in comparison to the remaining groups (Table 1 and Table 2) is further confirmed by the betaine stability (Table 3), as this metabolite is proved to show radio-protective properties [45]. Betaine is derived endogenously from the oxidation of choline (or exogenously from the dietary sources) [46]. Thus, its reduction in the plasma or serum could be related to the impaired choline oxidation in mitochondria [47].

In CAIR/CONV/SIB radiotherapy a decrease in creatinine was also observed (Table 2). In our previous study, we connected the decrease in the creatinine levels with an altered energy metabolism and/or lower muscle mass in the patients with high acute radiation-associated toxicity [9]. That is why no significant changes in creatinine are observed in case of the patients treated with Manchester RT, revealing a better overall status than the remaining patients. The reason why a decrease in the creatinine levels is not evident in CHRT may be the use of cisplatin-based chemotherapy—this drug is nephrotoxic and tends to increase the creatinine levels [48].

We identified seven metabolites significantly altered exclusively in CHRT (Figure S4): isoleucine, leucine and valine, as well as glycine, tyrosine, methanol and glucose. Thus, these metabolites seem to be a biomarker primarily of the chemotherapy effects. In the CHRT schedule, chemotherapy was administered at weeks 0, 3 and 6 (depending on how many cycles were scheduled for a patient). One week after the administration of the first and the second cycle of chemotherapy, we observed a short-term increase in the blood serum branched-chain amino acids (isoleucine, leucine and valine) (Table 1). It is claimed that after administration of cisplatin the BCAA levels in the blood/serum could be reduced due to the nephrotoxicity of the drug [49]. The increased contribution of the plasma BCAAs to the biomass in the tumors relying on the BCAA metabolism for growth is also expected to reduce the systemic BCAA levels [50]. In turn, their elevations are reported in some cancers with the decreased utilization of the circulating BCAAs, which is due to the increased tissue protein breakdown [51]. Such observations were confirmed in the animal studies [52]. Furthermore, Cobo-Dols et al. [53] (using laboratory amino acid analyzer) showed a significant increase in 17 of 27 different blood serum amino acids after the first cycle of cisplatin-based chemotherapy treatment for lung or head and neck cancers. Additionally, they reported increases in other amino acids, like alanine, glutamine, glycine and tyrosine [53]. In our study these amino acids are also increased in the CHRT group (except alanine, which is decreased) (Table 1). It should be noted that the tissue levels of the branched chain amino acids measured with HR MAS 1H NMR were detected as higher in both HNSCC and lymph-node metastatic tissues than in the normal tissue [54], which implies also the higher levels of BCAAs in the blood. Unfortunately, apart from ours there are no NMR studies dealing with the serum levels of BCAAs in this cancer. One also should bear in mind that some amino acids, e.g., glycine, are involved in cytoprotection and regulated cell death, and thus they may be useful in the therapy monitoring [55]. From the cited papers a general conclusion emerges that the amino acids are a valuable marker of the increased protein turnover, but there is still not a definitive explanation of such processes. In HNSCC the amino acids-related metabolic signature may be used to explain the metabolic profile modification after CHT/CHRT and in therapy monitoring.

Finally, we analyzed the statistically important metabolites in terms of their correlation with the volumes receiving a particular dose of radiation. To the best of our knowledge this is the first metabolomic study of such an effect with regard to a particular RT modality—which means that the alterations in the metabolic profiles are analyzed for the homogenous groups of patients treated with the same modality.

Two different effects can be analyzed based on the selected irradiated volumes. The volume receiving 70 Gy (or 50 Gy in Manchester RT) corresponds approximately to the planning treatment volume (PTV); thus, it reflects the primary tumor size plus margin. The metabolic alterations showing a significant correlation with this volume reflect the tumor metabolic response to the treatment. As the dose decreases, the irradiated volume depends less and less on the size of the primary tumor and the observed metabolic effects reflect mainly the irradiation of normal tissue.

Although the correlations are sparse and only in few cases the R value is > 0.5, some preliminary conclusions can be drawn. The most frequent correlations with the volume receiving the maximum dose are observed in BCAAs (CHRT and CAIR/CONV/SIB) and, to a lesser extent, in NAG/CRP (CAIR/CONV/SIB) (Figure 4 and Figure 5). The alterations in the BCAA signals are also correlated with the volumes receiving lower doses of radiation (Figure 4, Figure 5 and Figure 6). Thus, this is probably a common metabolic response of the primary tumor and the normal tissue to irradiation. There are also some discrepancies in the correlations of the inflammatory markers (NAG and CRP). Though, as mentioned earlier, a significant positive correlation between NAG and CRP can be observed [12], both markers presumably reflect different aspects of the inflammatory response [27,28]. In CHRT CRP shows a significant correlation with the volume of the irradiated normal tissue (Figure 4). In CAIR/CONV/SIB radiotherapy NAG and CRP correlate only with the volumes receiving higher (50–70 Gy) doses (Figure 5), while no correlation was observed in Manchester RT (Figure 6). Though the mechanism of glycation in human disease is not yet fully understood, NAG (or GlycA) is more and more often recognized as better at reflecting the degree of systemic inflammation than CRP, since it integrates more inflammatory pathways. It is also claimed to be a more stable marker, of a lower intra-individual variability than CRP—its levels are similar in both serum and plasma samples, in fasting and non-fasting states, and also after a short or long-term storage [56].

Similar dependencies on the modality are observed for the lymphocyte count. Lymphocytes are sensitive to irradiation [57] and this is reflected in the lower lymphocyte count during CHRT and CAIR/CONS/SIB than during hypo-fractionated Manchester RT (51 Gy in 17 fractions). Although, the only significant negative correlation with the irradiated volume was observed during the last three weeks of CAIR/SIB/CONV radiotherapy (for the volumes receiving from 5 to 60 Gy) (Figure 5). In Manchester radiotherapy the strong negative correlations of the irradiated volumes with the levels of glutamine, glycine and lipids (at 3.2 ppm) are observed in week-1 (Figure 6). However, OPLS-DA shows no significant metabolic alterations due to RT in the first two weeks for this treatment modality (Table 3). In spite of the thorough search of the data, nothing has been found to explain these correlations, both in terms of the methodological flaws and the biological explanation. More unexpected correlations were observed at week-4, where the lipid signals (at 0.9, 1.3 and 5.3 ppm) were positively correlated with the volumes irradiated with the doses in the range of 10–40 Gy. Although the overall lipid signals in the Manchester method decreased during the course of RT, the patients with the larger irradiated areas did indeed experience a slight increase in the lipid levels in the last week of the treatment. Many factors may be responsible for the increased serum lipids, e.g., diabetes, infections, cardiovascular diseases, but in this particular case we were not able to identify any of such connections with the patients’ clinical data.

Based on the presented results, the size of the irradiated volume (within a particular treatment modality) has a minor impact on the observed changes of the blood serum metabolites.

In summary, the blood serum metabolic profiles of the HNSCC patients are significantly affected by the anticancer radio- or chemoradiotherapy. The observed metabolic alterations intensify with the progression of the therapy, but the changes are too slow to be markedly visible at the weekly intervals. However, the comparison of the consecutive blood serum metabolic profiles with the baseline profile detected for the sample, taken immediately before the therapy, provides the significant metabolic markers of the response to therapy. In case of chemoradiotherapy, it was possible to distinguish a separate response pattern characteristic to chemotherapy. These metabolic changes were observed one week after administration of the first and the second cycle of CHT. Some of the observed disturbances in the metabolic profiles were correlated with the irradiated volumes; however, these correlations were weak or at best moderate. This may indicate that for such locations of the tumor, as in case of HNSCC and within a given radiotherapy modality, the differences in the irradiated volumes are too small to exert an effect on the overall metabolic response to radiotherapy.

## 5. Conclusions

NMR-based metabolomics was successfully employed in the studies of the “real-time” metabolic response to radio- and chemoradiotherapy of head and neck cancers. The changes in the metabolic profile of the blood serum were identified as corresponding to the systemic and tumor-related responses to the therapy: inflammation, disturbed glyoxalase system, altered energy metabolism as well as altered lipid profile and choline-related signaling. The metabolites associated with a secondary response, i.e., dysphagia and weight loss, have also been identified. The disturbances in amino acids metabolism (especially in BCAAs) are supposed to be characteristic for chemotherapy.

The blood serum metabolic profile seems to be slightly dependent on the irradiated volume, and such dependence is mainly limited to the inflammatory markers (positively correlated) and lymphocytes (negatively correlated with the irradiated volume).

The presented metabolomic approach gives insight into the systemic response to the anticancer treatment, with a possible distinction of the response of the normal tissue and the tumor.

## Figures and Tables

**Table 1:** Results from the OPLS-DA analysis of the blood serum metabolic profile changes occurring during concurrent chemoradiotherapy (CHRT).

**Weeks of CHRT Treatment**		**0 vs. 1**	**0 vs. 2**	**0 vs. 3**	**0 vs. 4**	**0 vs. 5**	**0 vs. 6**	**0 vs. 7**
**OPLS-DA Model Quality Parameters**								
R^2^X		0.05	0.05	0.1	0.06	0.06	0.06	0.06
R^2^		0.49	0.54	0.5	0.61	0.7	0.69	0.71
Q^2^		0.33	0.35	0.34	0.54	0.56	0.61	0.63
**Number of orthogonal components**								
		2	2	2	2	3	2	2
**Cumulative R^2^X of orthogonal components**								
R^2^X(o)		0.66	0.59	0.57	0.6	0.67	0.59	0.59
**Metabolites increased during radiotherapy**								
	ppm	p(corr)	p(corr)	p(corr)	p(corr)	p(corr)	p(corr)	p(corr)
Isoleucine	0.95	0.4			0.4			
Leucine	0.977	0.56			0.35			
		0.64			0.41			
		0.56			0.41			
Valine	1.005	0.7			0.49			
		0.62			0.46			
Isoleucine	1.02	0.59			0.51			
Valine	1.055	0.48			0.42			
		0.54			0.4			
Lipids	1.3				>0.4		>0.45	>0.5
Low ppm slope								
NAG	2.057	0.43		0.4	0.6	0.43	0.53	0.59
N-acetylcysteine	2.089	0.53		0.38	0.58	0.41	0.47	0.53
Glutamine	2.14–2.17	>0.45			>0.38			
Creatinine	3.05	0.45						
Glycine	3.57			0.54	0.6	0.57	0.6	0.52
Glycerol	3.6			0.52	0.58	0.54	0.6	0.6
				0.48	0.6	0.58	0.63	0.63
Valine	3.62	0.54						
Glycerol	3.68			0.4		0.5	0.52	0.6
				0.36	0.4	0.47	0.15	0.49
				0.55	0.7	0.52	0.47	0.65
				0.56		0.57	0.58	0.59
Glycolate	3.96			0.66	0.72	0.66	0.64	0.65
Tyrosine	6.92	0.44						
Tyrosine	7.21	0.4						
**Metabolites decreased radiotherapy**								
Lipids Low ppm slope	0.9	>0.4	>0.4	>0.6	>0.65	>0.65	>0.71	>0.73
Alanine	1.45					0.37	0.51	
Lipids	3.2	>0.45	>0.5	>0.6	>0.65	>0.67	>0.67	>0.64
Low ppm slope								
Betaine	3.28							0.39
Methanol	3.38			0.41		0.5		0.43
Glucose	5.2						0.36	

**Table 2:** Results from the OPLS-DA analysis of the blood serum metabolic profile changes occurring during radiotherapy of the patients receiving CAIR/CONV/SIB fractionated radiotherapy (RT).

**Weeks of CAIR/CONV/SIB Treatment**		**0 vs. 3**	**0 vs. 4**	**0 vs. 5**	**0 vs. 6**
**OPLS-DA Model Quality Parameters**					
R^2^X		0.145	0.062	0.07	0.09
R^2^		0.56	0.63	0.66	0.7
Q^2^		0.29	0.48	0.43	0.56
**Number of orthogonal components**					
		2	2	2	2
**Cumulative R^2^X of orthogonal components**					
R^2^X(o)		0.51	0.59	0.59	0.57
**Metabolites increased during RT**					
	ppm	p(corr)	p(corr)	p(corr)	p(corr)
NAG	2.057	0.31	0.43	0.58	0.38
N-acetylcysteine	2.089	0.32	0.43	0.57	0.37
Glycerol	3.6	0.39	0.51	0.6	0.56
		0.37	0.48	0.63	0.49
Glycerol	3.68	0.31	0.48	0.51	0.48
		0.3	0.44	0.41	0.41
		0.43	0.58	0.58	0.58
		0.49	0.51	0.65	0.57
Glycolate	3.96	0.48	0.62	0.74	0.58
**Metabolites decreased during RT**					
Lipids Low ppm slope	0.9	>0.45	>0.5	>0.47	>0.62
Lipids	1.3	>0.44		0.34	0.34
Alanine	1.45				0.46
Creatinine	3.05		0.44	0.45	0.57
Lipids Low ppm slope	3.2	>0.5	0.54	>0.49	>0.62
Betaine	3.28		0.44		0.33
Lipids	5.3	>0.4			>0.35

**Table 3:** Results from the OPLS-DA analysis of the blood serum metabolic profile changes occurring during radiotherapy of the patients receiving Manchester fractionated radiotherapy (RT).

**Weeks of Manchester Treatment**		**0 vs. 3**	**0 vs. 4**
**OPLS-DA Model Quality Parameters**			
R^2^X		0.07	0.1
R^2^		0.75	0.79
Q^2^		0.57	0.63
**Number of orthogonal components**			
		3	2
**Cumulative R^2^X of orthogonal components**			
R^2^X(o)		0.77	0.7
**Metabolites increased during RT**			
	ppm	p(corr)	p(corr)
Lysine	1.7		0.48
NAG	2.057	0.39	0.4
N-acetylcysteine	2.089	0.44	0.43
Glutamine	2.14–2.17		0.39
Lysine	3.02		0.45
Glycerol	3.6	0.61	0.5
		0.62	0.53
Glycerol	3.68	0.54	0.45
		0.54	0.47
		0.58	0.51
		0.63	0.51
Glycolate	3.96	0.69	0.57
**Metabolites decreased during RT**			
Lipids Low ppm slope	0.9	>0.35	>0.57
Lipids	1.3	>0.32	>0.34
Lipids Low ppm slope	3.2	>0.43	>0.6

## Data Availability

The data presented in this study are available on request from the corresponding author. The data are not anonymized and thus not publicly available.

## Supplementary Materials

The following are available online at https://www.mdpi.com/article/10.3390/ijms22126310/s1.
