# Supplementary material for: NMR-Based Metabolomics in Investigation of the Radiation Induced Changes in Blood Serum of Head and Neck Cancer Patients and Its Correlation with the Tissue Volumes Exposed to the Particulate Doses
Source: Int J Mol Sci. 2021 Jun 11;22(12):6310. doi: 10.3390/ijms22126310 (PMC8231285; doi:10.3390/ijms22126310)
Supplement: Supplementary file 1 [file ijms-22-06310-s001.zip › ijms-1229801-supplementary.pdf]

Supplementary material for:

NMR-Based Metabolomics in Investigation of the Radiation Induced Changes in Blood Serum of Head and Neck Cancer PA-Tients and Its Correlation with the Tissue Volumes Exposed to the Particulate Doses

Łukasz Boguszewicz [1,*], Agata Bieleń [2], Mateusz Ciszek [1], Jacek Wendykier [3], Krzysztof Szczepanik [4], Agnieszka Skorupa [1], Jolanta Mrochem-Kwarciak [5], Krzysztof Składowski [2] and Maria Sokół [1]

1 Department of Medical Physics, Maria Sklodowska-Curie National Research Institute of Oncology, Gliwice Branch, 44-102 Gliwice, Poland; mateusz.ciszek@io.gliwice.pl (M.C.); agnieszka.skorupa@io.gliwice.pl (A.S.); maria.sokol@io.gliwice.pl (M.S.)

2 1st Radiation and Clinical Oncology Department, Maria Sklodowska-Curie National Research Institute of Oncology, Gliwice Branch, 44-102 Gliwice, Poland; agata.bielen@io.gliwice.pl (A.B.); krzysztof.skladowski@io.gliwice.pl (K.S.)

3 Radiotherapy Planning Department, Maria Sklodowska-Curie National Research Institute of Oncology Gliwice Branch, 44-102 Gliwice, Poland; jacek.wendykier@io.gliwice.pl

4 Radiotherapy Department, Maria Sklodowska-Curie National Research Institute of Oncology Gliwice Branch, 44-102 Gliwice, Poland; krzysztof.szczepanik@io.gliwice.pl

5 Analytics and Clinical Biochemistry Department, Maria Sklodowska-Curie National Research Institute of Oncology Gliwice Branch, 44-102 Gliwice, Poland; jolanta.mrochem-kwarciak@io.gliwice.pl

* Correspondence: lukasz.boguszewicz@io.gliwice.pl

Table S1. Characteristics of the studied groups

RT fractionation		No. of patients	Median age	Sex		TNM			
				M	F	I	II	III	IV
CHRT	2 Gy per fraction, 35 fractions, total dose 70 Gy, delivered once-a-day and 5-days-a-week with weekend break, for 7 weeks	55	58 (41-79)	43	12		3	12	40
CAIR	1,8 Gy per fraction, 40 fractions, total dose 72 Gy, delivered once-a-day and 7-days-a-week, for 6 weeks	22	63 (46-77)	17	5		2	14	6
CONV	2 Gy per fraction, 35 fractions, total dose 70 Gy, delivered once-a-day and 5-days-a-week with weekend break, for 7 weeks	7	60 (48-76)	3	4	1	2	1	3
SIB	2.2 Gy per fraction to total dose 66 Gy for gross tumor volume (PTV1), 2.0 Gy per frac-tion to total dose 60 Gy for gross tumor volume plus anatomical margins (PTV2), 1.8 Gy per fraction to total dose 54Gy for elective fields (PTV3), all in 30 fractions, deliv-ered once-a-day and 5-days-a-week with weekend break, for 6 weeks	3	67 (56-77)	0	3	1		1	1
Manchester	3 Gy per fraction, 17 fractions, total dose 51 Gy, delivered once-a-day and 5-days-a-week with weekend break, for 3.5 weeks	19	65 47-79)	16	3	18	1		
Total		106	63 (41-79)	79	27	20	8	28	50

The characteristics of the acquired spectra as well as the pulse sequence parameters

- NOESY (Nuclear Overhauser Effect Spectroscopy) – an overview of all types of molecules.
- CPMG (Carr-Purcell-Meiboom-Gill) - information on only low molecular weight metabolites.
- DIFF (diffusion edited) - mainly macromolecular signals.
- Two dimensional (2D) JRES (J-resolved) – visualization of scalar couplings and improved metabolite identification.

Table S2. NMR pulse sequence parameters.

Pulse program	**NOESYGPPR1D**	**CPMGPR1D**	**LEDBPGPPR2S1D**	**JRESGPPRQF**
**TD**	65536	65536	65536	8192
**SW** [ppm]	30	20	30	16.62
**AQ** [sec]	2.73	4.09	2.73	0.62
**D1** [sec]	4	4	4	2
**D8** [sec]	0.01	-	-	-
**D16** [sec]	-	-	0.0002	0.0002
**D20** [sec]	-	0.0003	0.12	-
**D21** [sec]	-	-	0.005	-
**DS**	4	4	4	16
**L4**	-	126	-	-
**NS**	32	64	64	1
**DELTA1** [sec]	-	-	0.11572488	-
**DELTA2** [sec]	-	-	0.004172	-

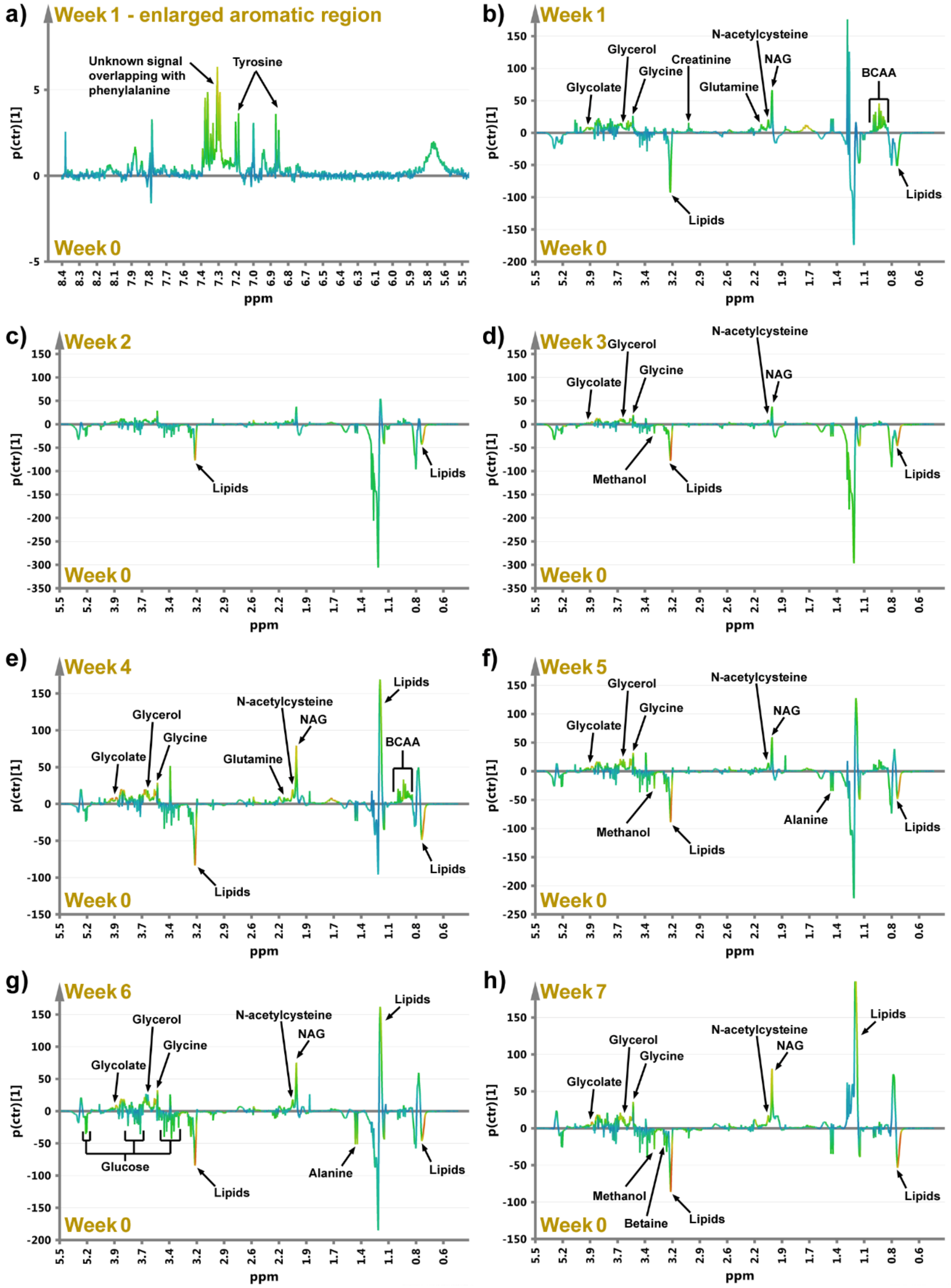

Figure S1. OPLS-DA s-line plots identifying metabolites important for differentiation of week-0 from the consecutive weeks of the CHRT treatment. These plots are complementary to the OPLS-DA scores plots in Figure 2 in the manuscript. Week-0 vs week-1 (aromatic region of NMR spectrum) (a), week-1 (b), week-2 (c), week-3 (d), week-4 (e), week-5 (f), week-6 (g) and week-7 (h).

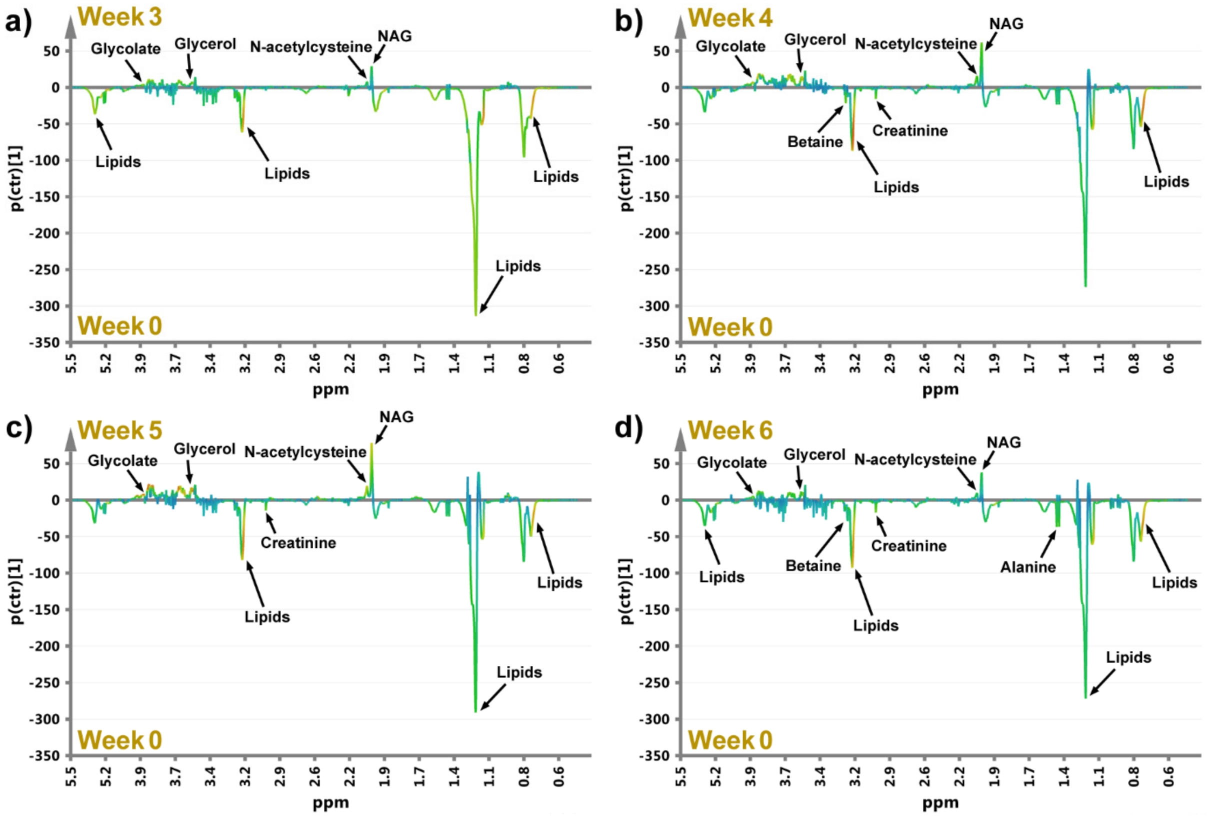

Figure S2. OPLS-DA s-line plots identifying metabolites important for differentiation of week-0 from the consecutive weeks of the CAIR/CONV/SIB treatment. These plots are complementary to the OPLS-DA scores plots in Figure 3 in the manuscript. Week-0 vs week-3 (a), week-4 (b), week-5 (c), week-6 (d).

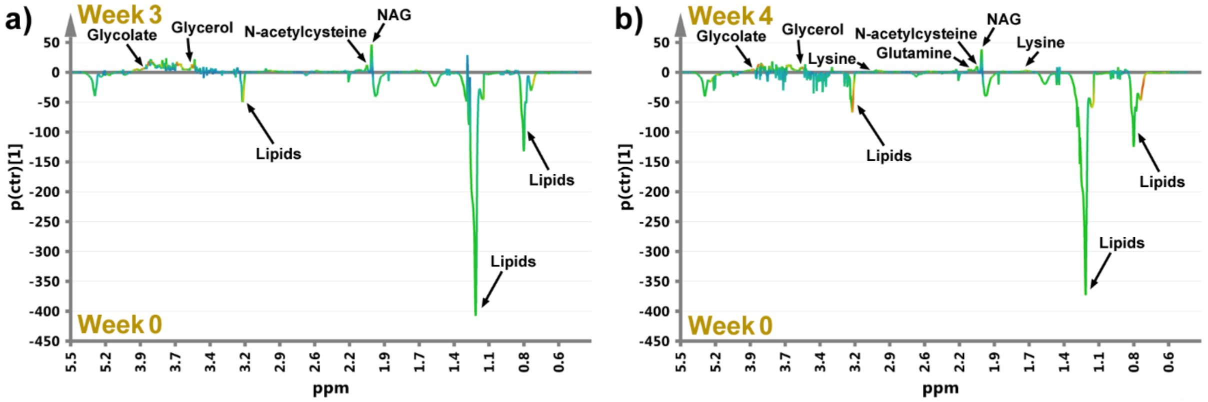

Figure S3. OPLS-DA s-line plots identifying metabolites important for differentiation of week-0 from the week-3 and week-4 of the Manchester treatment. These plots are complementary to the OPLS-DA scores plots in Figure 4 in the manuscript. Week-0 vs week-3 (a), week-4 (b).

Treatment method	CHRT	CAIR/CONV/SIB	Manchester
No. of important OPLS-DA models	7	4	2
**Metabolites:**	**No. of models where the metabolite was important**		
Alanine	2	1	
BCAA: isoleucine	2		
BCAA: leucine	2		
BCAA: valine	2		
Betaine	1	2	
Creatinine	1	3	
Glucose	1		
Glutamine	2		
Glycerol	5	4	2
Glycine	5		
Glycolate	5	4	2
Lip 0.9	7	4	2
Lip 1.3	3	3	2
Lip 3.2	7	4	2
Lip 5.3		2	2
Lysine			1
Methanol	3		
N-acetylcysteine	6	4	2
NAG	6	4	2
Tyrosine	1		

Figure S4. Summary of the alterations in the blood serum metabolic profiles due to various treatment modalities. The numbers denote in how many of the OPLS-DA models the metabolite was identified as significant for discrimination between week-0 and other, consecutive weeks of treatment. Metabolites highlighted in green are significantly affected only by the CHRT. Dark yellow indicates metabolites significantly changed in all the three treatment groups. The remaining metabolites are highlighted in gray.
